# A Post-Implanto-Prosthetic Rehabilitation Study Regarding the Degree of Improvement in Patients’ Quality of Life: A Before–After Study

**DOI:** 10.3390/healthcare12141378

**Published:** 2024-07-10

**Authors:** Cosmin Ionuț Lixandru, Ionela Maniu, Maria Mihaela Cernușcă-Mițariu, Mihai Iulian Făgețan, Ioan Sebastian Cernușcă-Mițariu, Horațiu Paul Domnariu, George Adrian Lixandru, Carmen Daniela Domnariu

**Affiliations:** 1 Faculty of Medicine, “Lucian Blaga” University, 550024 Sibiu, Romania; maria.mitariu@ulbsibiu.ro (M.M.C.-M.); mihai.fagetan@ulbsibiu.ro (M.I.F.); sebastian.mitariu@ulbsibiu.ro (I.S.C.-M.); george.lixandru@ulbsibiu.ro (G.A.L.); carmen.domnariu@ulbsibiu.ro (C.D.D.); 2 Mathematics and Informatics Department, Faculty of Sciences, Research Center in Informatics and Information Technology, “Lucian Blaga” University, 550024 Sibiu, Romania; 3 Research Team, Pediatric Clinical Hospital Sibiu, 550166 Sibiu, Romania; 4 Doctoral School of Medicine, University of Oradea, 410087 Oradea, Romania; domnariu.horatiupaul@student.uoradea.ro

**Keywords:** implanto-prosthetic rehabilitation, dental implant, quality of life, questionnaire, Oral Health Impact Profile-14 (OHIP-14)

## Abstract

Background: Implant–prosthetic rehabilitation has the ability to improve the quality of life of patients, because, in addition to the role of restoring masticatory function, they also have many other benefits, such as restoring aesthetics or improving speech. This study aimed to analyze whether patients’ quality of life was improved by implanto-prosthetic rehabilitation and which were the most important aspects. Materials and Methods: In this before–after study, we applied the Oral Health Impact Profile (OHIP-14) questionnaire to analyze the degree to which complex implanto-prosthetic rehabilitation led or not to an increase in patients’ quality of life. The present study was carried out at the level of a private medical center in a city in the central region of Romania and included patients who visited this medical center between January and June 2022 and who benefited from a complex implanto-prosthetic rehabilitation, with the total number of patients eligible for inclusion in the study being 116. Results: Overall, an improvement in quality of life after implant-prosthetic rehabilitation was found. Patients’ gender, age, or educational level did not significantly influence their responses. The network analysis offered an overview (intuitive visual representation) of the similarities but also the differences in the OHIP-14 item relationships in both situations: before and after oral rehabilitation. Conclusions: A better understanding of how patients perceive implanto-prosthetic rehabilitation and the aspects that influence this perception can lead to an improvement in their quality of life, increasing the addressability of this type of medical procedure.

## 1. Introduction

The loss of teeth and the appearance of so-called edentacy represent an irreversible phenomenon with multiple consequences. Edentation represents a state of oral health with significant effects on the psychological, aesthetic, social and psychological states of patients. Currently, there are a number of therapeutic approaches used to solve it, ranging from the use of conventional prostheses to complex implanto-prosthetic rehabilitation; therefore, it is very important to monitor patients’ perceptions of the chosen therapeutic solution. There are a number of transversal studies and longitudinal ones that have compared the perceptions of patients who benefit from conventional prostheses and the perceptions of patients who benefit from complex fixed implanto-prosthetic rehabilitation [1,2].

Endo-osseous implants (EI), introduced more than 40 years ago, have seen an exponential increase in their use in implanto-prosthetic rehabilitation. They are now the standard of care, providing predictable and reliable treatment options for the rehabilitation of edentulous or partially edentulous patients, as well as those with congenitally absent teeth [3]. The absence of bilateral maxillary lateral incisors (MBMLI) presents both aesthetic and functional challenges, requiring a multidisciplinary approach to treatment. When MBMLI occurs without the absence of other teeth apart from the third molars, it is classified as mild hypodontia. Patients with MBMLI often seek orthodontic treatment with high aesthetic expectations due to the anterior location of the malocclusion [4]. Moreover, the lack of upper incisors poses a significant problem and requires complex treatment. Treatment options for missing upper incisors include orthodontic space closure, resin-bonded bridges, osseointegrated implants, removable partial dentures and the auto-transplantation of developing premolars [5]. The optimal solution currently used to correct these anomalies is implant rehabilitation, because any possible damage to the adjacent teeth is avoided, leading to the optimal achievement of the occlusion [5]. Implanto-prosthetic rehabilitation is an essential branch of modern dental medicine, offering advanced and sustainable solutions for the restoration of dental functionality and aesthetics. This innovative technique involves the use of dental implants to replace missing or damaged teeth, offering patients an effective and long-lasting alternative to traditional dentures. One of the main advantages of dental implants is their ability to restore masticatory functionality to an optimal level [6]. In addition, the literature also highlights the reduction of discomfort in eating certain foods, improving nutrition and contributing to the health of the jaw bone and gums. Other benefits include improving the patient’s psychological state and increasing their quality of life [7]. By replacing missing teeth, implants provide a solid anchor for dental crowns or bridges, allowing patients to chew and bite with the same efficiency as with natural teeth. This increased functionality contributes to improving the quality of nutrition, as well as avoiding digestive problems associated with ineffective mastication [6]. Among the benefits of dental implants are also their ability to prevent bone atrophy, their versatility and their ability to improve the aesthetic appearance of the smile [6]. The multiple advantages that implanto-prosthetic rehabilitation confers are one of the main reasons underlying the continuous increase in the number of requests for these procedures from patients, according to official statistics [8]. The preoperative examination is an essential pillar in the process of planning and implementing dental implants. This crucial phase allows the surgeon to gain a detailed insight into the patient’s health, identify potential risks and develop a personalized treatment plan [9].

Implanto-prosthetic rehabilitation, in addition to the many advantages that it confers, can also present a series of risks, such as the occurrence of peri-implantitis, which can cause failure in implanto-prosthetic therapy [1]. Among the risks of the surgical procedure are bleeding after the operation, numbness in the case of mandibular nerve damage, possible infections and even the failure of the osseointegration of the implants [7]. Areas of tissue deficiency should be treated by grafting prior to surgery, but these aspects are often neglected. One of the greatest disadvantages is that the use of implants requires training, but this is not sufficiently addressed in the training of future practitioners, which is why additional training is needed in this field [10]. Among the factors on which the success of implanto-prosthetic therapy depends are the general state of health of the patient, the degree of maintenance of good oral hygiene, the anatomical peculiarities, the existence or not of a periodontal pathology, the appropriate restoration of the occlusal morphology and the maintenance of sterility conditions at the time of the surgical intervention, as well as the number of implants applied or the professional training of the surgeon [1].

The concept of patients’ quality of life is an essential field in health and medical care, reflecting the general state of well-being of the individual in the context of their health conditions. This concept transcends medical aspects and includes psychological, social and spiritual dimensions, having a significant impact on the patient’s experience during treatment and recovery. The concept of quality of life is notable for its subjectivity, individual variation, complex interplay of factors, evolution over time and underlying meaning [11]. The concept of quality of life (QOL) is a notion that can be perceived differently by patients, being closely related to the type of population studied, with the possibility to vary depending on the social, cultural, political or practical context. In the literature [11,12], quality of life is seen as the subjective satisfaction experienced by a person and which is projected by this person to all aspects of their life, including physical, psychological, social and spiritual aspects. A deterioration in health, whether it is irreversible or not, as well as limited mobility problems, lead to the appearance of disabilities in the performance of daily activities, thus influencing quality of life in a negative way [11,12].

Oral-health-related quality of life (OHQoL) is influenced by the impact that different oral health problems have on patients’ daily activities, being an important indicator of the quality of oral rehabilitation services. This is an important component of the Global Oral Health Program developed at the level of the World Health Organization and can be assessed with the help of OHIP-type questionnaires. Evaluating the degree of improvement in patients’ quality of life as a result of different therapeutic approaches can indicate the degree of effectiveness of the treatments applied from the patient’s point of view. The use of various oral health assessment tools, such as the Oral Health Impact Profile (OHIP-14, OHIP-EDENT) or the Oral Health Impact Profile (GOHAI), can offer information on patient satisfaction in relation to their oral health [2]. The OHIP-14 questionnaire has proven to be a questionnaire with satisfactory reliability and validity and is successfully used in the evaluation of the quality of life of patients who are referred for implanto-prosthetic therapy or oral rehabilitation with the help of conventional dental prostheses [13].

The study of the quality of life of implanto-prosthetic rehabilitated patients represents an essential component in evaluating the success and impact of these procedures on the general well-being of individuals. The goal of this study is to understand to what extent implanto-prosthetic rehabilitation influences the quality of life of patients (the null hypothesis is that there is no difference in quality of life before and after this procedure) and to identify the factors (and their interrelations) that contribute to this influence.

## 2. Materials and Methods

The present study was a before–after study that was conducted between January and June 2022, including a group of 116 patients. The patients included in this study were patients of a private clinic in the city of Sibiu, who benefited from implanto-prosthetic rehabilitation. The participants in this study were aged between 20 and 70 years. This study was approved by the Scientific Research Ethics Committee of the “Lucian Blaga” University of Sibiu through ethical approval number 34 from 16 November 2023. The criteria for inclusion in the study were (a) patients over 20 years old; (b) patients who had given their consent regarding participation in the study; (c) patients who had not benefited from implanto-prosthetic rehabilitation in the past; (d) patients who had missing teeth for more than a year before presenting to the office; (e) patients who did not have general contraindications for the insertion of dental implants. The exclusion criteria for the study were (a) patients under 20 years of age; (b) patients who did not give their consent regarding participation in the study; (c) patients who had benefited from implanto-prosthetic rehabilitation in the past; (d) patients who had missing teeth for less than a year before presenting to the office; (e) patients who had absolute contraindications regarding the insertion of dental implants. Patients’ consent regarding their participation in the study was represented by the voluntary completion and return of the OHIP-14 questionnaire.

In this regard, the OHIP-14 questionnaire was used. The initial form of the Oral Health Impact Profile (OHIP) is based on a questionnaire of 49 items and represents one of the most used tools for the measurement, from a subjective point of view, of the state of oral health among patients. Among its advantages, it can highlight, both in terms of frequency and severity, the different oral health problems reported by patients. Over time, attempts have been made to develop shorter forms of the OHIP questionnaire, such as the OHIP-14 or OHIP-EDENT, because the initial version with a set of 49 questions is difficult to apply in certain studies and also provides more of an overall evaluation of the level of oral health that is not always necessary and relevant in the study of a specific subject [1,14]. An important aspect of the OHIP-14 questionnaire is its structure, which divides the questions into seven distinct domains—pain, discomfort, dietary restrictions, emotional discomfort, social discomfort, functional disability and inability to perform daily responsibilities—in order to determine to what extent the quality of life of the patients included in the study has improved [13,15]. The questionnaire was administered before the surgical intervention and 2 months after the implanto-prosthetic rehabilitation. 

Data analysis was performed using the Statistical Package for the Social Sciences (SPSS v.20, IBM Corp., Armonk, NY, USA) and R v.4.0.5 software (the R Foundation for Statistical Computing, Vienna, Austria). We calculated the mean OHIP scores for the entire sample, to provide a general picture of the level of impact on their quality of life. To assess significant changes before and after the implanto-prosthetic intervention, we calculated the mean difference in the OHIP scores before and after treatment for each patient. The standard deviation, a measure of the dispersion of the data, was also computed for the OHIP scores before and after treatment. This was useful to assess the degree of variability in patient responses. In addition, a 95% confidence interval was used for the mean difference, thus providing an idea of the precision of the estimate. We also calculated the effect size to assess the practical magnitude of the difference. *p*-values for differences were determined using the Wilcoxon signed-rank test, and a *p*-value < 0.05 was considered statistically significant. Furthermore, network analysis, a multivariate data analysis method, was performed to investigate the relationship between the OHIP items and groups of items, before and after oral rehabilitation. Network nodes represented the questionnaire items while edges represented the partial correlations between the items (the edge thickness was proportional to the strength of the association/relationship).

## 3. Results

In order to analyze the results of the research, a total of 116 questionnaires completed by patients who benefited from implanto-prosthetic rehabilitation services were analyzed. The first step in this regard was the analysis of the socio-demographic characteristics of the patients included in the study. Thus, the study showed that, of the total number of patients included in the study, more than half were women, representing a percentage of 61.21% of the total, while 38.79% of the participants were men. The mean age of the respondents was 44.41 years, with most patients falling within the 30 to 55 age range. Regarding the place of origin of the participants in the study, 67.24% of them lived in the urban area, while only 32.76% of them lived in the rural area. In terms of the educational level of the patients included in this research, 64.66% of them had graduated from higher education and 35.34% had graduated from secondary education. Table 1 presents a comparison of the different aspects of the OHIP-14, studied before and after oral rehabilitation. As can be seen, for many patients, the difficulties in pronouncing words had significantly decreased following oral rehabilitation (39.66 rarely, 17.24% never). At the same time, a significant decrease also occurred regarding the deterioration of taste (29.31% had this problem rarely, while 19.83% never had this problem after oral rehabilitation), the level of pain (37.07% felt pain after implantation, and 19.83% never felt strong pain after oral rehabilitation), discomfort regarding eating (33.62 rarely felt any discomfort while eating, and 17.24% never felt any discomfort while eating), the feeling of insecurity or stress or difficulties in relaxation. However, we noticed that, even after oral rehabilitation, some patients felt irritable, or there was an inability to perform certain activities after oral rehabilitation. One of the most important aspects that we observed was that, for many patients, their life satisfaction increased after oral rehabilitation.

**Figure 1:**
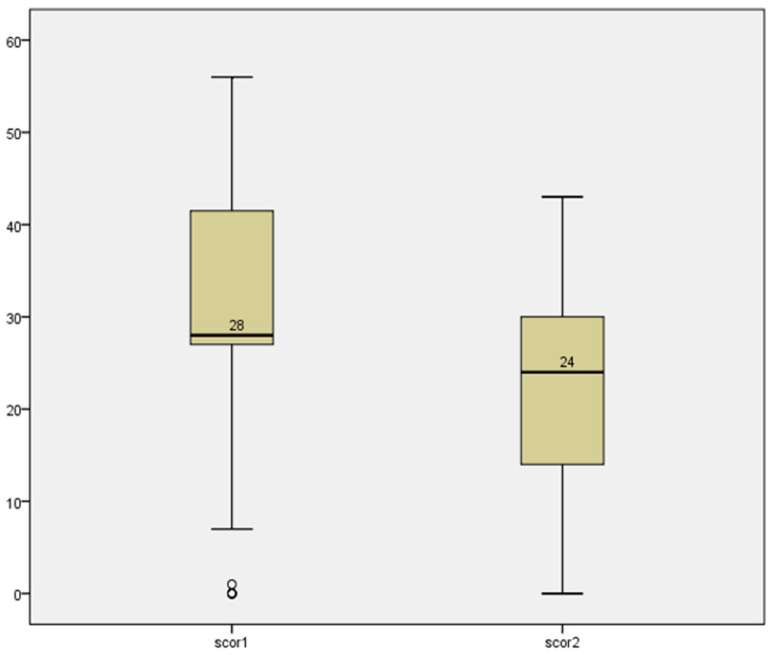
OHIP score before and after oral rehabilitation (Wilcoxon signed-rank test, *p* = 0.000).

Based on the analyzed data, the OHIP-14 score decreased from 29.64 (SD = 12.121) at the first evaluation to 22.18 (SD = 11.274) at the second evaluation. An effect size of 0.98 suggested an improvement in oral-health-related quality of life (Figure 1). This reduction was statistically significant for all seven domains of the scale. For domains D5–D7, the size of the effect was moderate (0.58–0.59), with a larger effect being observed for domains 1 and 2.

Moreover, a comparative analysis of the OHIP scores was carried out between the categories of the following factors: age, gender, place of residence and completed studies. In the case of both measurements (before/after oral rehabilitation), in terms of gender, women had a slightly higher score than men (before: *p* = 0.334, after: *p* = 0.045). Age did not have a significant influence on patient satisfaction; no significant difference was observed between patients from urban areas and those from rural areas (before: *p* = 0.629, after: *p* = 0.262); and no difference was found between patients with secondary education and those with higher education (before: *p* = 0.332, after: *p* = 0.912).

**Figure 2:**
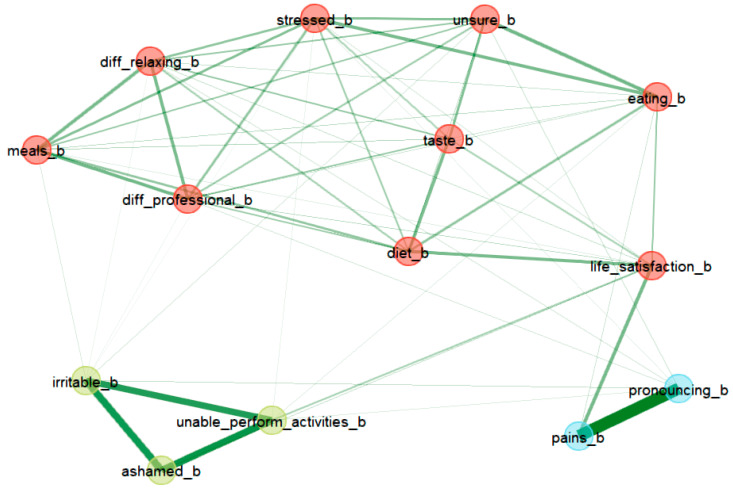
Network analysis of OHIP items before oral rehabilitation.

**Figure 3:**
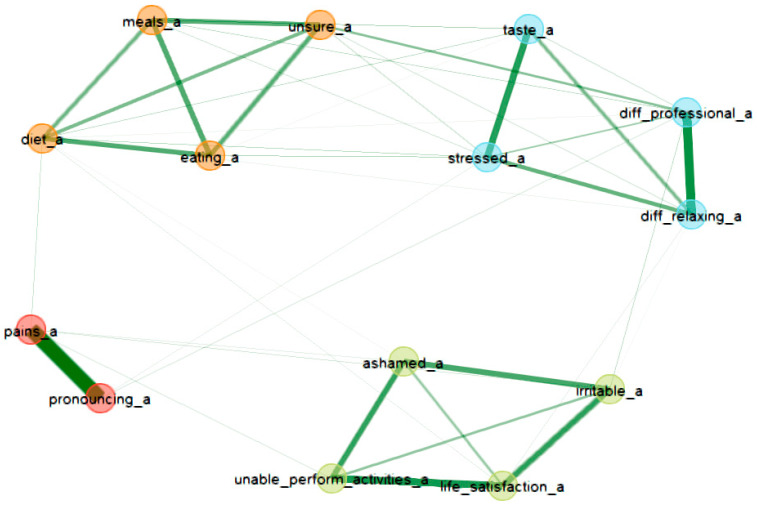
Network analysis of OHIP items after oral rehabilitation.

Figure 2 and Figure 3 present the relationships between the OHIP items before and after oral rehabilitation. The nodes of the network represent the questionnaire items, while the edges represent their associations (partial correlations). Strong associations were observed between strong pain before/after implantation and difficulties in pronouncing words before/after implantation and feeling irritable/ashamed/unable to perform certain activities before/after implantation. We can observe, in the period before implantation, a denser network between items related to physical/psychological disability/discomfort and life satisfaction, while, in the period after implantation, the network is sparser, being able to distinguish two different communities/clusters: one including items related to meals/eating/diet and the other including items related to stress/professional difficulties/relaxation difficulties.

## 4. Discussion

The primary goal of oral rehabilitation is to improve the oral health status and quality of life of patients, considering that the absence of teeth has negative consequences, influencing psychosocial and functional aspects. Overall, the results of our data analysis suggest a statistically significant and clinically relevant improvement in oral-health-related quality of life, as indicated by the decrease in the OHIP-14 scores and the substantial effect size. The OHIP-14 score initially was 29.64 (SD = 12.12) during the first evaluation, while, in the second evaluation, the score decreased to 22.18 (SD = 11.27). This indicates a reduction in the OHIP-14 score, suggesting an improvement in oral-health-related quality of life. A significant improvement in the quality of life of patients who benefited from implanto-prosthetic rehabilitation was also found in the study conducted by Bugone et al. This reinforces the idea that the observed change in the OHIP-14 scores was not merely due to chance and suggests a substantial improvement in oral-health-related quality of life [16].

In our study, the reduction was found to be statistically significant for all seven domains of the scale. This implies that the observed changes in the scores were unlikely to have occurred by random chance alone. A larger effect was detected for domains 1 and 2 compared to domains D5–D7. This suggests that the impact of the intervention or change was more substantial in domains 1 and 2, indicating potential areas for improvement or greater sensitivity to the intervention. An improvement in quality of life after implanto-prosthetic rehabilitation was also highlighted in the study carried out by Raes and colleagues; the main aspects that improved after rehabilitation were, as in our study, the pronunciation of words, the perception of taste, a reduction in pain, improved comfort and also a reduction in the feeling of insecurity [17,18]. Moreover, other studies in this field have revealed results similar to those of our study [19,20]. Although the results of our analysis, at the level of the entire scale, are similar to those in the literature, differences can be observed at the item level. In addition to the univariate analytical methodologies, the network analysis approach offers an overview (an intuitive representation) of the most significantly related variables and provides information on the ways in which different items influence each other. The two networks also offer the possibility to visualize the similarities but also differences in the item relationships in both situations: before and after oral rehabilitation

Based on the information provided by our participants, there were no statistically significant differences observed between men and women, urban and rural patients and people with secondary and higher education in both measurements, except for the second measurements in that the higher score reported by women compared to men was statistically significant. These results are consistent with those of the study conducted by Sargolzaie et al., which also revealed an improvement in patients’ quality of life after implanto-prosthetic rehabilitation—in particular, a reduction in functional limitations and pain, as well as psychological and social discomfort [21]. Moreover, the results of our study are in agreement with the results of the study carried out by Petricevic and colleagues, according to which the level of improvement in quality of life following implanto-prosthetic rehabilitation was similar between women and men [22]. Patient gender did not significantly influence the reported results in the study by Niakan et al. [23]. With regard to the quite significant differences in the area of residence, we believe that this difference stemmed from the fact that people in rural areas do not tend to seek medical help when they have a dental problem, probably because of barriers to access to medical services. Likewise, other studies in the field, such as those carried out by Erkapers and colleagues and that of Furuyama and his colleagues, demonstrate the fact that age does not represent an impact factor on the satisfaction of implanto-prosthetic rehabilitated patients [24,25,26]. This aspect was also revealed in the study by Lee et al., as well as in the study by Niakan et al. [23,27]. Despite this, our study contradicts other studies in the field by Campos [28] and Kuoppala [29], according to which older patients reported better quality of life compared to younger patients.

A review of the literature by Boven et al., examining the masticatory progress, bite force, satisfaction and nutritional status of implanto-prosthetic rehabilitated patients, revealed a high level of patient satisfaction with comfort, although this was not always accompanied by a general improvement in quality of life. According to the authors, implanto-prosthetic therapy improves the efficiency of and satisfaction with masticatory function and increases the maximum bite force, but the effects on quality of life remain uncertain [30]. The results of our study are similar to those of the study of Alzarea, which determined that the health-related quality of life improved after oral rehabilitation, regarding aspects such as pronouncing words, the sense of taste, the pain level, difficulties when eating, feeling tense, an unsatisfactory diet, the interruption of meals, difficulties in relaxing, feeling embarrassed and feeling irritable [1]. Moreover, the study carried out by McKenna and his collaborators found that, following implanto-prosthetic rehabilitation, there were decreases in areas such as functional limitations, physical pain, physical discomfort, social disability and psychological disability, which is consistent with the results found in our study [31].

Implanto-prosthetic rehabilitation has a number of advantages, such as the restoration of masticatory function and nutrition support, benefiting the health of the jaw bone and gums and even reducing the psychological stress caused by tooth loss and improving quality of life in general. Among the benefits of dental implants are their ability to prevent bone atrophy, their versatility and their ability to improve the aesthetic appearance of the smile [6,7]. At the same time, among the primary disadvantages of implanto-prosthetic rehabilitation, studies in the field highlight the following: the occurrence of peri-implantitis, the risk of bleeding following the intervention, numbness due to possible damage to the mandibular nerve or the failure of osseointegration [1,7,10]. In the study by Jamilian et al., the main disadvantage reported was that even if the implant was inserted after the age of 19 years, the adjacent teeth and surrounding alveolar bone may continue to grow vertically and erupt, which may lead to infra-occlusion in implant restoration [5]. This means that a gap may occur between the gingival margin of the implant restoration and that of the adjacent natural teeth after several years of treatment, causing the implant to become sunken [5]. Pre- and post-surgical planning should be established before the intervention. The success of implanto-prosthetic rehabilitation depends considerably on the competence of dentists specializing in oral surgery, as well as on the quality of their collaboration with dentists specializing in dental prosthetics. In most cases, the prosthetist is not consulted until after the surgical intervention, which compromises the outcome of prosthetic rehabilitation [32].

The first limitation of this study concerns the sample size. A sample of 116 patients can be considered relatively small and may influence the representativeness of the results. At the same time, there is the possibility that other factors, which were not taken into account, may have influenced the results. Moreover, the OHIP-14 questionnaire involves subjective answers from patients. Their responses may vary due to the subjective interpretation of the questions and individual variations in expressing their health status. The expansion of the sample could strengthen the validity and representativeness of the results, providing a more comprehensive picture of the impact of implanto-prosthetic rehabilitation on quality of life. Moreover, investigating the long-term effects of implanto-prosthetic rehabilitation could provide valuable information about the durability of the benefits and possible changes over time. At the same time, beyond the OHIP-14 questionnaire, the addition of other objective or subjective measures of quality of life could provide a more complete perspective on the impact of implanto-prosthetic rehabilitation. The questionnaire was applied twice to patients who benefited from the implanto-prosthetic rehabilitation procedure. Another limitation of our study is the fact that the study did not evaluate the anatomical area in which the implanto-prosthetic rehabilitation was performed. In addition, we did not use approaches based on multivariate regression (including controlling for confounders), which provide an alternative method of analysis.

Thus, further research could provide more in-depth results regarding the evolution of the quality of life improvement in patients who have benefited from such rehabilitation. We believe that a more in-depth study in this field should take into account the different categories of factors that could influence patient satisfaction with implanto-prosthetic treatment. Moreover, a more comprehensive study should include patient groups that receive implants after tooth loss and patient groups that receive implants due to congenital missing teeth, taking into consideration the anatomical area where the implanto-prosthetic rehabilitation is performed.

## 5. Conclusions

This study showed that there was a significant improvement in patients’ quality of life after implanto-prosthetic rehabilitation. In this regard, we believe that oral health professionals should place greater emphasis on aspects related to improving the quality of life of their patients and on promoting implanto-prosthetic rehabilitation as a form of treatment that can lead to a considerable increase in the quality of life of patients. This can only be achieved by having an open discussion with the patient to understand the issues that they are facing due to their dental problems, physically, psychologically and socially.

## Figures and Tables

**Table 1:** Comparison of the problems faced by patients before and after oral rehabilitation.

Item	Code	M ± SD	Mean Diff (95%CI) *p*
Difficulties in pronouncing words before the implant	D1_b(pronouncing_b)	2.09 ± 0.94	0.647 (0.493; 0.800) 0.000
Difficulties in pronouncing words after the implant	D1_a(pronouncing_a)	1.44 ± 1.02	
Deterioration of taste before the implant	D2_b(taste_b)	2.20 ± 0.97	0.629 (0.477; 0.782) 0.000
Deterioration of taste after the implant	D2_a(taste_a)	1.57 ± 1.11	
Strong pains before the implant	D3_b(pains_b)	2.14 ± 0.92	0.741 (0.592; 0.891) 0.000
Strong pains after the implant	D3_a(pains_a)	1.40 ± 1.02	
Discomfort in eating food before the implant	D4_b(eating_b)	2.20 ± 0.92	0.698 (0.554; 0.842) 0.000
Discomfort in eating food after the implant	D4_a(eating_a)	1.50 ± 0.99	
Feeling unsure before the implant	D5_b(unsure_b)	2.16 ± 0.94	0.655 (0.505; 0.805) 0.000
Feeling unsure after the implant	D5_a(unsure_a)	1.51 ± 1.05	
Feeling stressed before implant	D6_b(stressed_b)	2.17 ± 0.93	0.586 (0.437; 0.736) 0.000
Feeling stressed after implant	D6_a(stressed_a)	1.59 ± 1.10	
Unsatisfactory diet before the implant	D7_b(diet_b)	2.19 ± 0.98	0.741 (0.594; 0.889) 0.000
Unsatisfactory diet after the implant	D7_a(diet_a)	1.45 ± 0.99	
Interruption of meals before the implant	D8_b(meals_b)	2.17 ± 0.95	0.681 (0.534; 0.828) 0.000
Interruption of meals after the implant	D8_a(meals_a)	1.49 ± 1.00	
Difficulty relaxing before the implant	D9_b(diff_relaxing_b)	2.16 ± 0.94	0.603 (0.460; 0.747) 0.000
Difficulty relaxing after the implant	D9_a(diff_relaxing_a)	1.56 ± 1.07	
Feeling ashamed before the implant	D10_b(ashamed_b)	1.96 ± 1.01	0.181 (−0.006; 0.368) 0.060
Feeling ashamed after the implant	D10_a(ashamed_a)	1.78 ± 1.07	
Feeling irritable before the implant	D11_b(irritable_b)	1.95 ± 1.03	0.172 (−0.008; 0.353) 0.061
Feeling irritable after the implant	D11_a(irritable_a)	1.78 ± 1.14	
Difficulties in professional activities before the implant	D12_b(diff_professional_b)	2.17 ± 0.94	0.595 (0.456; 0.734) 0.000
Difficulties in professional activities after the implant	D12_a(diff_professional_a)	1.58 ± 1.04	
Life satisfaction before the implant	D13_b(life_satisfaction_b)	2.11 ± 0.92	0.388 (0.212; 0.564) 0.000
Life satisfaction after the implant	D13_a(life_satisfaction_a)	1.72 ± 1.09	
Unable to perform certain activities before the implant	D14_b(unable_perform_activities_b)	1.97 ± 1.03	0.138 (−0.047; 0.322) 0.137
Unable to perform certain activities after the implant	D14_a(unable_perform_activities_a)	1.83 ± 1.13	

“b” denotes “before”; “a” denotes “after”.

## Data Availability

The data are available on request from the corresponding author.
